# Change Fatigue, Job Crafting and Associated Factors Among Nurses: A Multicentre Cross‐Sectional Study

**DOI:** 10.1002/nop2.70503

**Published:** 2026-03-31

**Authors:** Xinyue Zhang, Ying Wang, Yue Guo, Shiyu Yin, Zijing Huang

**Affiliations:** ^1^ Department of Nursing, Tongji Hospital, Tongji Medical College Huazhong University of Science and Technology Wuhan Hubei China; ^2^ School of Nursing, Tongji Medical College Huazhong University of Science and Technology Wuhan Hubei China

**Keywords:** change fatigue, job crafting, nurses, nursing management

## Abstract

**Aim:**

To explore the current level of change fatigue among nurses in Hubei Province, China and to examine its correlation with job crafting and other influencing factors.

**Design:**

A multicentre cross‐sectional online survey among 22,030 nurses from 79 hospitals across 17 cities in Hubei Province.

**Methods:**

In January 2024, a self‐designed general information questionnaire, change fatigue scale and job crafting scale were used to collect data. Descriptive statistics, univariate analyses and multiple linear stepwise regression analyses were performed using IBM SPSS 26.0 and R 4.4.1 software for data analysis.

**Results:**

A total of 21,588 valid questionnaires were collected, resulting in a response rate of 97.99%. Nurses had a change fatigue score of 23.62 (SD = 7.98), which was negatively correlated with job crafting and its three dimensions (all *p* < 0.001). Income satisfaction, health condition, relational crafting, age, cognitive crafting, type of hospital, professional title, experience of changing employment and task crafting were predictors of nurses' change fatigue, collectively explaining 27.00% of the total variance.

**Conclusion:**

Both change fatigue and job crafting among nurses were moderately high, and they exhibited a negative correlation. Nurses experiencing poor job crafting, dissatisfaction with income and health, older age, employment in non‐tertiary hospitals, holding intermediate titles and having job‐changing experiences were more susceptible to change fatigue.

**Relevance to Clinical Practice:**

With the ongoing reform of the medical system, there is a growing need for increased attention to nurses' change fatigue. Nursing managers should pay more attention to change fatigue among nurses and actively identify its influencing factors, and implement organisational readiness strategies, including securing resource support and enhancing communication and feedback mechanisms, to mitigate the negative influence of change.

**Reporting Method:**

Study methods and results were reported in accordance with the STROBE checklist.

**Patient or Public Contribution:**

No patient or public contribution. All data information came from the nurses.

## 1 Introduction

Driven by multiple demands, including innovation, technological optimisation, quality improvement and fiscal austerity, health care institutions are increasingly required to realign their priorities in service delivery, resource allocation and organisational management (Beaulieu et al. 2023; Brown et al. 2018). As integral members of the medical service team, nurses are highly sensitive to organisational change. When organisational change becomes routine, it often results in a series of negative influences on clinical nurses, such as change fatigue, diminished job satisfaction, heightened stress, increased burnout rates and turnover rates, endocrine disorders, sleep disturbances, etc. and even influence patient care outcomes (Barker and Nussbaum 2011; Bernerth et al. 2011; Brown et al. 2018; De Vries and de Vries 2023; Dossey 2008; Dunleavy et al. 2003; McMillan and Perron 2020; McVicar 2016; Niu et al. 2011). Frequent organisational change can also induce a saturation effect. Before implementing new changes, nurses need to adapt to the pressure from the previous changes. When a period of recovery or transition after a change is not afforded, or a change pressure source beyond the nurse's self‐management ability is introduced before the recovery period, a saturation effect occurs (Bernerth et al. 2011; Ead 2015). Among the needs of organisational change and development, nurses' change fatigue has emerged as a topic deserving increasing attention in recent years. The Registered Nurses Association of Ontario (RNAO) proposed in 2011 that, to cope with the rapid changes in the healthcare system, efforts should be made to minimise nurses' excessive pressure and feelings of helplessness. Moreover, the focus regarding nurses' fatigue should shift towards describing the psychological impacts of rapid and frequent changes, rather than solely concentrating on behavioural performance. Previous studies have also indicated that nurses typically perceive mental fatigue as being more severe than physical fatigue (Barker and Nussbaum 2011).

Change fatigue is an overwhelming sense of stress, exhaustion and burnout associated with rapid and continuous change in the workplace (Brown and Abuatiq 2020; Camilleri et al. 2019; McMillan and Perron 2020). The concepts of ‘change fatigue’ and ‘excessive change’ are prevalent in the literature across various industries, highlighting the adverse outcomes of repeated or poorly managed change initiatives or changes implemented without the opportunity for recovery or stabilisation (Bernerth et al. 2011; Camilleri et al. 2019; Ead 2015). Change fatigue tends to develop gradually over time and exacerbate the existing pressure sources of change, resulting in symptoms of fatigue and burnout. This, in turn, impacts team commitment, employee well‐being and quality of patient care (Bernerth et al. 2011; Ead 2015; McMillan and Perron 2013; Smollan 2015). Studies indicate that nurses experience change fatigue at a rate three times higher than individuals in other roles within the healthcare system, and nurses who suffer from change fatigue often show apathy and passive acceptance, so they are also more likely to be ignored by managers and themselves (Sanders et al. 2013; Sarigul and Ugurluoglu 2023). Over time, this will not only adversely affect the physical and mental well‐being of the nurses, but also further hinder the reform and development of the hospital. Therefore, actively identifying and addressing nurses' change fatigue is of great practical significance.

Currently, many scholars in the nursing field have been actively investigating nurses' change fatigue, encompassing aspects such as its definition, causes, consequences, assessment tools, mitigation strategies and more (Beaulieu et al. 2023; Brown et al. 2018; Camilleri et al. 2019; McMillan and Perron 2020; Sarigul and Ugurluoglu 2023). However, the majority of relevant research in China concentrates on topics like nurses' burnout, fatigue, work pressure and similar areas, without yet introducing the concept of change fatigue and exploring it.

In China, Hubei Province, one of the most populous regions, has experienced an accelerated pace of healthcare system reform in recent years. These reforms involve not only the redistribution and optimisation of medical resources but also substantial changes in nurses' roles, responsibilities and work intensity. Such ongoing organisational changes may increase workload, task complexity and patient interaction, thereby influencing nurses' work experiences and career satisfaction. In addition, large‐scale public health emergencies, such as the coronavirus disease 2019 (COVID‐19) pandemic (Zhou et al. 2020; Du et al. 2020), have further intensified the frequency and magnitude of organisational changes faced by nurses. Although China has entered the post‐pandemic period, previous evidence suggests that the psychological and occupational impacts of pandemic‐related disruptions on nurses may persist over time, particularly in regions that experienced prolonged and intensive public health responses (Liu et al. 2022; Preti et al. 2020). Against this broader context of continuous healthcare reform and cumulative change‐related stress, nurses' change fatigue and work‐related adaptation warrant sustained attention (Carmassi et al. 2020; Li et al. 2021; Liu et al. 2023; Xiang et al. 2020). In this context, job crafting has received increasing attention as an adaptive work behaviour that enables employees to actively adjust their work in response to changing demands (Rudolph et al. 2017). It is commonly conceptualised as comprising three dimensions: task crafting, relational crafting and cognitive crafting (Golfenshtein et al. 2024). In nursing practice, these behaviours are reflected in nurses' proactive adjustments to tasks, interpersonal interactions and cognitive framing of work, with the aim of aligning job demands with their individual resources and needs (Iida et al. 2024). Therefore, this study aimed to investigate the current situation of nurses' change fatigue and job crafting in Hubei Province, explore the correlation between change fatigue and job crafting and analyse the associated factors of change fatigue, so as to provide practical basis and reference for alleviating nurses' change fatigue, optimizing occupational conditions and promoting the sustainable development of medical reform.

## 2 Methods

### 2.1 Design, Setting and Participants

From January 5 to January 12, 2024, a multicentre cross‐sectional survey was carried out in Hubei Province, China. A combination of stratified sampling and convenience sampling was used to investigate. Stratified sampling was conducted according to the quantity and capacity of hospitals across the 17 cities in Hubei Province to determine the number of hospitals and nurses surveyed in each city. Each city comprised a minimum of 3 tertiary hospitals and 2 secondary hospitals (according to the classification by the National Health Commission of the People's Republic of China). Finally, this study surveyed a total of 79 hospitals, comprising 40 tertiary hospitals, 36 secondary hospitals and 3 primary hospitals. Within each hospital, the convenience sampling method was used to survey the nurses across all departments.

The questionnaire hosted on the ‘WJX’ (Changsha Ranxing Information Technology Company) platform, along with the survey plan outlined in the invitation letter, was prepared by the researchers. These materials were then disseminated to the directors of the nursing department at each hospital, explaining the precautions and principles of questionnaire filling. Subsequently, the nursing department director sent the questionnaire to the head nurse of each department, who then distributed it to the nurses who met the inclusion criteria. The questionnaire was standardised with uniform instructions. Each participant was restricted to filling out the questionnaire once using a single electronic device, with a time limit of 10 min. Additionally, the survey's objectives, significance and the researchers' contact information were provided to facilitate prompt consultation for nurses with queries. Before filling in the questionnaire, the head nurse explained the guidelines for questionnaire completion again to the nurses.

The inclusion criteria of nurses were as follows: (a) being registered nurses in China, (b) having clinical nursing experience and (c) being at least 18 years old. Nurses on leave, out of position, on internship or pursuing further education were excluded.

Out of 22,030 nurses who participated in the survey, questionnaires containing incomplete, incorrect or clearly erroneous information were excluded. Consequently, 21,588 valid questionnaires were obtained, with a response rate of 97.99%.

### 2.2 Measures

The questionnaire consisted of three main parts.

- Self‐designed general information questionnaire: It included gender, age, marital status, number of children, religious beliefs, educational level, health condition, working years, professional title, position, type of hospital, city and full name of the hospital, department, income satisfaction and experience of changing employment. Among them, the city and full name of the hospital and the department were only used for data source documentation.
- Change fatigue scale: Bernerth et al. (2011) developed this scale in 2011, drawing upon stress and organisational change theories and employing the multi‐step approach described by Hinkin. Comprising 6 items, the scale utilises a Likert 7‐level scoring method, ranging from ‘strongly disagree’ to ‘strongly agree’, with scores ranging from 1 to 7. Higher scores indicate greater fatigue experienced by employees in response to organisational change. The original scale demonstrated a Cronbach's *α* coefficient of 0.850. Due to the lack of the Chinese version of the scale, after obtaining the authorisation of the original author, the Brislin translation model was used to translate, back‐translate and culturally adjust the original change fatigue scale. The Cronbach's *α* coefficient of the Chinese version of the scale was 0.918, the split‐half reliability was 0.911, the retest reliability was 0.894 and the indicators of validity were also within the acceptable range.
- Job crafting scale: Developed by Dvorak (2014) in 2014, based on the boundary shift theory. This study employed the Chinese version of the scale, adapted by Chinese scholar Zhu Shixiao (2019). The scale includes three dimensions: task crafting, cognitive crafting and relational crafting. Each dimension contains a total of 7 items, totalling 21 items. The items are rated on a 1–5 Likert‐type scale where ‘1 = disagree’ and ‘5 = very strongly agree’. Higher scores indicate a higher level of job crafting among nurses. The Cronbach's *α* coefficients of the three dimensions and overall scale of the original study were 0.84, 0.89, 0.89 and 0.91, respectively, which were 0.84, 0.89, 0.89 and 0.89 in this study.

### 2.3 Statistical Analysis

IBM SPSS Statistics 26.0 (IBM Corp., Armonk, NY, USA) (IBM Corp 2019) and R 4.4.1 software (R Core Team, Vienna, Austria) (R Core Team 2023) were utilised for data collation and analysis. Descriptive statistics were used to determine the distribution of general data and scale scores. Skewness and kurtosis were used to test normality (All variables were found to conform to a normal distribution in this study except for age and working years). Continuous variables with normal distribution were reported in the form of mean and standard deviation (SD), and continuous variables with skewed distribution were reported in the form of median and interquartile range (IQR). The categorical variables were expressed as frequency and composition ratio. Student's *t*‐test, one‐way ANOVA and Pearson correlation analysis were used for univariate analysis. For the results of *p* < 0.05 in one‐way ANOVA, Tukey's HSD method was further applied for multiple comparisons. Variables that were statistically significant in the univariate analyses were selected as candidate independent variables and entered into the stepwise multiple linear regression model. The stepwise method was applied with entry and removal criteria of *α*_in_ = 0.05 and *α*_out_ = 0.10. Ordered categorical variables, including health condition, type of hospital and income satisfaction, were treated as continuous variables to reflect the natural order of the categories. Health condition was coded from ‘very poor’ to ‘very good’ (1–5), type of hospital from ‘tertiary’ to ‘primary’ (1–3) and income satisfaction from ‘very dissatisfied’ to ‘very satisfied’ (1–5). The regression coefficients (B) represent the expected change in the change‐fatigue score for a one‐level increase in the corresponding variable. Additionally, multicollinearity of the multiple regression model was assessed, with tolerance values below 0.10 or variance inflation factor (VIF) above 10 indicating multicollinearity. A two‐tailed *p* value of < 0.05 was considered statistically significant.

### 2.4 Ethical Considerations

This study was approved by the Ethics Committee of Tongji Hospital, Tongji Medical College, Huazhong University of Science and Technology (No. TJ‐IRB202401089). The objectives and procedures were explained to the participants. They were assured that participation in the study was voluntary and anonymous and that refusing to participate would not have any impact on their work and life. All data collected is kept secure and confidential, accessible only to the research team.

## 3 Results

### 3.1 Characteristics of Hospitals and Participants

This study involved 21,588 nurses from 79 hospitals located across 17 cities in Hubei Province. Among these, there were 40 tertiary hospitals (*n* = 17,050, 78.98%), 36 secondary hospitals (*n* = 4432, 20.53%) and 3 primary hospitals (*n* = 106, 0.49%). These nurses covered almost all departmental specialties, with internal medicine and surgery being the main ones, accounting for approximately 25.44% and 23.03% of the total, respectively. Other major departments comprised obstetrics and gynecology, pediatrics, operating rooms and emergency departments.

Among the 21,588 nurses surveyed, the median age was 33 years (IQR: 19–37; range: 18–60; mean: 33.86) and the median years of experience were 10 years (IQR: 7–15; range: < 1–43; mean: 12.02). Female nurses accounted for 96.53% (*n* = 20,838) of the total and 76.23% (*n* = 16,456) were married. 82.16% (*n* = 17,737) of nurses held a bachelor's degree, while 95.05% had either junior or intermediate professional titles. Merely 3.26% (*n* = 703) of nurses reported having religious beliefs and 8.01% (*n* = 1730) perceived their health condition to be below average. Additionally, 16.00% (*n* = 3454) of nurses held administrative positions such as nurse manager and 17.54% (*n* = 3786) had experience with changing employment. Further details are provided in Table 1.

**TABLE 1:** Relationship between nurse characteristics and change fatigue.

Variables	*n* (%)	Change fatigue score (mean ± SD)	*t*/*F*	*p*
Gender				
Female	20,838 (96.53)	23.65 ± 7.96	2.326^a^	0.020*
Male	750 (3.47)	22.96 ± 8.56		
Marital status				
Married	16,456 (76.23)	23.68 ± 7.97	3.064^b^	0.027*
Unmarried	4659 (21.58)	23.35 ± 8.02		
Divorced	437 (2.02)	24.19 ± 7.99		
Widowed	36 (0.17)	24.72 ± 7.06		
Number of children				
0	6364 (29.48)	23.32 ± 8.02	4.821^b^	0.002**
1	10,957 (50.76)	23.77 ± 7.91		
2	4201 (19.46)	23.71 ± 8.07		
≥ 3	66 (0.31)	22.85 ± 8.61		
Religious beliefs				
Yes	703 (3.26)	24.07 ± 7.98	1.519^a^	0.129
No	20,885 (96.74)	23.61 ± 7.98		
Education level				
Junior college	3495 (16.19)	23.65 ± 7.69	0.528^b^	0.590
Undergraduate	17,737 (82.16)	23.63 ± 8.04		
Postgraduate and above	356 (1.65)	23.20 ± 7.90		
Health condition				
Very poor	150 (0.69)	30.41 ± 8.89	623.042^b^	< 0.001***
Poor	1580 (7.32)	28.18 ± 7.24		
Fair	11,024 (51.07)	24.94 ± 7.04		
Good	6618 (30.66)	22.01 ± 7.86		
Very good	2216 (10.26)	18.17 ± 9.09		
Type of hospital				
Tertiary	17,050 (78.98)	23.18 ± 8.14	129.948^b^	< 0.001***
Secondary	4432 (20.53)	25.27 ± 7.08		
Primary	106 (0.49)	26.60 ± 6.94		
Professional title				
Junior	10,304 (47.73)	23.41 ± 8.02	8.341^b^	< 0.001***
Intermediate	10,216 (47.32)	23.86 ± 7.95		
Associate senior and above	1068 (4.95)	23.48 ± 7.75		
Position				
Yes	3454 (16.00)	22.91 ± 7.77	5.719^a^	< 0.001***
No	18,134 (84.00)	23.76 ± 8.01		
Income satisfaction				
Very dissatisfied	708 (3.28)	30.16 ± 8.77	1769.144^b^	< 0.001***
Dissatisfied	2759 (12.78)	28.35 ± 6.37		
Neutral	10,736 (49.73)	25.16 ± 6.40		
Satisfied	5938 (27.51)	20.43 ± 7.76		
Very satisfied	1447 (6.70)	13.06 ± 7.06		
Experience of changing employment				
Yes	3786 (17.54)	24.71 ± 7.66	9.224^a^	< 0.001***
No	17,802 (82.46)	23.39 ± 8.03		

Abbreviation: SD, standard deviation.

^a^ Student's *t*‐test.

^b^ One‐way ANOVA test.

* *p* < 0.05.

** *p* < 0.01.

*** *p* < 0.001.

### 3.2 Nurses' Change Fatigue and Job Crafting

The scores of the Change Fatigue Scale and Job Crafting Scale were normally distributed. In this study, nurses scored an average of 23.62 (SD = 7.98) on the Change Fatigue Scale, with the highest scores observed for item 1 and item 6. The specific scores for each item are provided in Table 2. Nurses scored an average of 74.46 (SD = 14.01) on the Job Crafting Scale, with scores for task crafting, cognitive crafting and relational crafting dimensions at 22.57 (SD = 5.49), 25.50 (SD = 5.21) and 26.39 (SD = 5.14) points, respectively. It is noteworthy that the highest score was observed in relational crafting, whereas the lowest score was in task crafting (Table 2).

**TABLE 2:** Descriptive results of change fatigue and job crafting.

Variables	Range	Mean ± SD
Change fatigue	6–42	23.62 ± 7.98
1 Too many change initiatives are introduced in this organisation	1–7	4.02 ± 1.51
2 I am tired of all the changes in this organisation	1–7	3.54 ± 1.53
3 The amount of change that takes place in this organisation is overwhelming	1–7	3.68 ± 1.53
4 We are asked to change too many things in this organisation	1–7	3.82 ± 1.57
5 It feels like we are always being asking to change something around here	1–7	3.85 ± 1.58
6 I would like to see a period of stability before we change anything else in this organisation	1–7	4.71 ± 1.55
Job crafting	21–105	74.46 ± 14.01
Task crafting	7–35	22.57 ± 5.49
Cognitive crafting	7–35	25.50 ± 5.21
Relational crafting	7–35	26.39 ± 5.14

Abbreviation: SD, standard deviation.

### 3.3 Relationship Between Nurse Characteristics, Job Crafting and Change Fatigue

As shown in Table 1, in terms of personal factors, female nurses had a higher degree of change fatigue than men (*p* = 0.020) and the age of nurses was positively correlated with change fatigue (*p* < 0.001). Nurses with different marital status had different levels of change fatigue (*p* = 0.027), with unmarried nurses showing the lowest levels of change fatigue. The number of children also influenced nurses' change fatigue (*p* = 0.002). Specifically, nurses with 1 child had higher change fatigue than those without children (*p* = 0.002). Significant differences were observed in the level of change fatigue among nurses with different health conditions (*p* < 0.001). Multiple comparisons indicated that change fatigue scores differed across all health condition groups (all *p* ≤ 0.01), suggesting that better health status was associated with significantly lower change fatigue scores. There was no significant difference in the scores of change fatigue among nurses with different religions and education levels (*p* > 0.05). Regarding situational factors, there was a positive correlation between longer nursing work time and higher levels of change fatigue (*p* < 0.001). There were differences in the degree of change fatigue among nurses in different types of hospitals (*p* < 0.001). Subsequent analysis revealed no significant difference in change fatigue between secondary and primary hospitals (*p* = 0.201). However, change fatigue was significantly lower in tertiary hospitals compared to both secondary and primary hospitals (both *p* < 0.001). Nurses with intermediate professional titles exhibited higher levels of change fatigue compared to those with junior professional titles (*p* < 0.001). Additionally, nurses without positions experienced higher levels of change fatigue than those with positions (*p* < 0.001). In terms of income satisfaction, both single‐factor analysis and multiple tests showed significant differences in change fatigue levels among nurses with different income satisfaction (all *p* < 0.001), that is, with the increase of income satisfaction, the degree of change fatigue of nurses was lower. In addition, nurses who had changed their jobs had significantly higher levels of change fatigue (*p* < 0.001).

Pearson correlation analysis results (Table 3) showed that nurses' job crafting was negatively correlated with change fatigue (*r* = −0.316, *p* < 0.001). In addition, task crafting, cognitive crafting and relational crafting were also negatively correlated with change fatigue (all *p* < 0.001).

**TABLE 3:** Pearson correlation analysis between nurse characteristics, job crafting and change fatigue.

Variables	1	2	3	4	5	6	7
1. Age	1						
2. Working years	0.968***	1					
3. Change fatigue	0.034***	0.033***	1				
4. Job crafting	0.086***	0.084***	−0.316***	1			
5. Task crafting	0.008	0.008	−0.246***	0.840***	1		
6. Cognitive crafting	0.104***	0.100***	−0.296***	0.924***	0.647***	1	
7. Relational crafting	0.121***	0.118***	−0.300***	0.892***	0.567***	0.814***	1

*** *p* < 0.001.

### 3.4 Multiple Linear Regression Analysis of Change Fatigue

Multiple linear stepwise regression results showed that income satisfaction (*β* = −0.369, *p* < 0.001), health condition (*β* = −0.130, *p* < 0.001), Relational crafting (*β* = −0.077, *p* < 0.001), age (*β* = 0.040, *p* < 0.001), cognitive crafting (*β* = −0.052, *p* < 0.001), type of hospital (*β* = 0.032, *p* < 0.001), professional title (*β* = 0.042, *p* < 0.001), experience of changing employment (*β* = −0.024, *p* < 0.001) and task crafting (*β* = −0.025, *p* = 0.001) were predictors of nurses' change fatigue. The full model explained 27.0% of the variance in change fatigue (*R* ^2^ = 0.270, adjusted *R* ^2^ = 0.270; *F* = 887.766, *p* < 0.001). No multicollinearity was observed (Table 4).

**TABLE 4:** Multiple linear regression analysis results regarding the prediction of change fatigue.

Predictors	Unstandardised coefficient		Standardised coefficient (*β*)	*t*	*p*	95% CI^a^		Collinearity statistics	
	*B*	SE				Lower	Upper	Tolerance	VIF
Constant	42.769	0.444	—	96.231	< 0.001***	—	—	—	—
Age	0.043	0.008	0.040	5.086	< 0.001***	0.027	0.060	0.558	1.791
Health condition	−1.299	0.065	−0.130	−20.073	< 0.001***	−1.425	−1.172	0.806	1.240
Type of hospital	0.612	0.113	0.032	5.430	< 0.001***	0.391	0.833	0.949	1.054
Professional title	0.565	0.106	0.042	5.344	< 0.001***	0.358	0.772	0.560	1.786
Income satisfaction	−3.383	0.063	−0.369	−54.015	< 0.001***	−3.506	−3.261	0.726	1.377
Experience of changing employment	−0.505	0.123	−0.024	4.092	< 0.001***	−0.747	−0.263	0.978	1.023
Task crafting	−0.036	0.011	−0.025	−3.207	0.001**	−0.058	−0.014	0.567	1.763
Cognitive crafting	−0.079	0.017	−0.052	−4.721	< 0.001***	−0.112	−0.046	0.284	3.521
Relational crafting	−0.120	0.016	−0.077	−7.593	< 0.001***	−0.151	−0.089	0.326	3.063

*Note:* Model statistics: *R* ^2^ = 0.270, adjusted *R* ^2^ = 0.270; *F* = 887.766, *p* < 0.001; AIC = 144,151.6, BIC = 144,239.4.

Abbreviations: CI, confidence interval; SE, standard error; VIF, variance inflation factor.

^a^ The 95% CIs presented correspond to the unstandardised regression coefficient (*B*).

** *p* < 0.01.

*** *p* < 0.001.

To examine the contribution of different dimensions of job crafting, we included relational, cognitive and task crafting as separate variables in the regression model. Compared to the baseline model, which included only demographic and job‐related control variables (*R* ^2^ = 0.254, adjusted *R* ^2^ = 0.254; *F* = 1227.000, *p* < 0.001), the full model incorporating the three job crafting dimensions showed a modest improvement in explanatory power (Δ*R* ^2^ = 0.016, ΔAdjusted *R* ^2^ = 0.016). Model fit indices also supported this improvement: AIC decreased from 144,609.8 to 144,151.6 and BIC decreased from 144,673.6 to 144,239.4, indicating that the inclusion of job crafting dimensions slightly improved model fit, although their overall contribution to *R* ^2^ remained modest.

## 4 Discussion

Although related constructs such as burnout and job stress have been widely examined among Chinese nurses, empirical evidence focusing specifically on change fatigue remains limited. This study therefore extends existing research by examining change fatigue and its correlates in a large, multicentre nursing sample. The results showed that the change fatigue score among these 21,588 nurses was 23.62 ± 7.98 points. Compared with the score range of the questionnaire, it was higher than the median. It can be considered that the overall level of change fatigue among nurses is moderately high. This score surpassed the change fatigue scores reported for American nurses (Brown et al. 2018) in previous studies, likely influenced by cultural disparities, values and differences in healthcare systems, as well as experiencing the combined effects of COVID‐19. Given that this survey did not address COVID‐19‐related issues, it is challenging to substantiate this speculation. Chinese nurses may face heightened work pressure in their professional settings due to factors such as an aging population and rising disease burden. Consequently, they may be more prone to experiencing change fatigue. Moreover, with the ongoing reform and evolution of the Chinese healthcare system, nurses may be required to adapt to continuously shifting job demands and processes, thereby potentially exacerbating their levels of change fatigue. Considering cultural background, in China, nurses may tend towards a team‐oriented approach, prioritizing collective interests over individual achievements. This cultural context may lead nurses to place more emphasis on the impact of team‐based changes over their personal levels of change fatigue. Even if they experience fatigue, they may continue to support changes. In addition, insufficient support from the healthcare system or a lack of democracy in decision‐making processes, such as lack of appropriate training, poor communication, unsatisfactory working conditions and ‘bureaucratisation’ of decision‐making processes, can lead to nurses being overwhelmed, showing high levels of resistance, fatigue or maladjustment in the face of change. The item scores, particularly item 6, provide insight into nurses' expectations and needs during the transitional period of change. Therefore, based on ensuring the availability of a certain transition buffer, nursing managers can actively implement resilience training (Brown and Abuatiq 2020; Rogerson et al. 2016), mindfulness interventions (Muir and Keim‐Malpass 2020), assign accountability and adopt the change calendar to coordinate and manage workloads (Valusek 2007) in order to alleviate nurses' change fatigue, and in the establishment of a stable and equal communication mechanism, to broadly incorporate the suggestions and opinions of frontline clinical nurses, to be aware of the inequalities that may exist in the decision‐making for change, increasing readiness for change in the organisation.

In the results, we found that nurses' overall job crafting and scores on the dimensions were also in the middle to high range. However, these scores were all negatively correlated with change fatigue, that is, as nurses' change fatigue increased, their job crafting abilities (including task crafting, cognitive crafting and relational crafting) decreased. Similarly, this may also mean that the worse nurses are at crafting their work, the more likely they are to experience change fatigue, with the two showing a negative cycle. Notably, the strongest correlation was observed with relational crafting. Importantly, these findings indicate that nurses who are more successful in proactively crafting their work tend to experience more positive work‐related feelings and lower levels of burnout, highlighting the potential value of fostering job crafting behaviours as a strategy to mitigate change fatigue. Job crafting is the process by which employees reconstruct their jobs by changing job task, cognitive and relational boundaries (Zhang and Parker 2019), placing greater emphasis on emphasizing subjective initiative and motivation, which leads to a greater congruence between their intrinsic motivation and extrinsic behaviour. In the short term, job crafting can help employees adapt to work changes, improve work status quo and improve the match between people and jobs; in the long term, job crafting can enhance the sense of meaning and self‐efficacy, enrich work resources and improve work commitment and performance (Petrou et al. 2018; Rudolph et al. 2017; Tims et al. 2013). Initial facilitative job crafting can have positive effects, all of which increase the likelihood that they will continue job crafting, however negative experiences can inhibit this likelihood and lead directly to employee absenteeism, turnover, etc. (Grant and Ashford 2008). This study represents an initial exploration of nurses' job crafting behaviours and contributes to our understanding of its negative association with change fatigue. Therefore, it is crucial to recognise change fatigue and job crafting situation among nurses and to help nurses cope with change and enhance their job crafting skills by positively improving their internal and external environments. Externally, there is a need to ensure that nurses receive appropriate training to adapt to the new task requirements and cognitive demands of change. Moreover, proactive provision of psychological support is necessary to help them deal with stress, fatigue and other adverse emotions that may arise during periods of change. Adequate resource support, including time, manpower and material resources, is essential for nurses to effectively adapt to new job requirements and changes. Internally, nurses' work status and adaptability should be regularly assessed and through timely communication, management can explain to nurses the reasons, purpose and importance of the change, thereby enhancing their understanding and support for the change and reducing uncertainty and anxiety. Furthermore, fostering teamwork can improve nurses' productivity and job satisfaction, facilitating relational crafting and mitigating the negative impact of change.

This study also identified several influencing factors associated with nursing change fatigue. General factors included type of hospital, age and professional title. The level of change fatigue among nurses in tertiary hospitals was significantly lower compared to those in secondary and primary hospitals. This may be due to the fact that tertiary hospitals also usually imply more adequate resource conditions, more mature and efficient management systems and a more open and supportive organisational climate with greater professional autonomy for nurses. Therefore, nursing managers in primary and secondary hospitals should pay more attention to nurses' change fatigue, learn from higher‐level hospitals about management experiences, prepare for organisational change and improve the success rate and effect of change. This has been indicated in previous studies (Adams and Bond 2000; Brown et al. 2018), in which nurses in Magnet‐accredited hospitals reported higher job satisfaction. Positive hospital work environments are associated with greater control over the practice environment, use of systems of care that promote accountability and continuity of care and so on (Adams and Bond 2000). Unfortunately, there are currently no Magnet‐accredited hospitals in Hubei Province, preventing direct assessment of the effects of Magnet accreditation in this study. In this study, change fatigue among nurses was positively correlated with age, that is change fatigue was more pronounced at older ages. This result is contrary to the results of previous studies, which suggested that older nurses might possess more experience in dealing with change (Moore et al. 1996; Stensaker and Meyer 2012), while newly graduated nurses were more susceptible to change fatigue (Vestal 2013). This may be due to a combination of factors such as personal life factors as well as the work environment and culture, where older nurses may tend to be more conservative and stable and it may be more difficult for them to accept the uncertainty and instability that comes with change. In contrast, younger nurses may be more motivated and enthusiastic about new challenges and opportunities. Therefore, the characteristics and needs of nurses of different age groups need to be taken into account when designing change management strategies to better cope with change fatigue. Furthermore, higher age often corresponds to higher positions, increased responsibilities, expectations and pressures. Nurses with higher professional titles may need to coordinate and communicate with multiple stakeholders, including management, other healthcare staff and patients. This complex communication and coordination process may increase their workload and fatigue, confirming the correlation between job title and nurses' change fatigue. Thus, greater emphasis should be placed on the division of responsibilities and incentive policies to reduce the pressure for change.

Some individual related factors such as income satisfaction, health condition and experience of changing employment can also affect change fatigue among nurses. The negative correlation between change fatigue and job satisfaction (Brown et al. 2018; Kuokkanen et al. 2009; Teo et al. 2013) has been demonstrated in many previous studies, similar to the results of this study. This may be due to a combination of factors such as pay stability, job satisfaction, income stability in relation to pressure for change and pay as a motivating factor. Similar to this factor, health condition was also a protective factor against change fatigue in nurses, that is, good health contributes to an individual's ability to cope with change and stress and may also have a higher level of productivity and resilience, leading to greater ease and emotional stability in the face of change. In contrast, nurses in poorer health may be more susceptible to change and to physical and mental fatigue. Rather than large‐scale policy changes, feasible health‐supportive measures may include unit‐based stress management resources, peer support mechanisms and timely access to occupational health consultation during periods of intensive organisational change (Camilleri et al. 2019). Nurses who have experienced job changes were more prone to change fatigue, a point that this study wanted to explore based on the current context of high turnover among nurses. Each job change requires adaptation to a new work environment, team culture, work processes, etc. This frequent adaptation process increases the challenge of nurses' adaptability to change and reduces their confidence and sense of stability, and may be due to the lack of a sense of belonging and a support system, and the questioning of the stability of the work environment may make the nurses more sensitive and worried in the face of change and these emotional states may also increase their risk of change fatigue. Thus, under the premise of ensuring a stable and fair organisational climate, attention should be paid to the adaptability, change of mindset and career development planning of these nurses and necessary support and assistance should be provided to them to enhance their sense of identity and stability at work. It should be noted that many sources of change fatigue among Chinese nurses are rooted in broader systemic healthcare reforms, which are beyond the direct control of nursing managers. Therefore, the recommendations proposed in this study primarily target modifiable organisational processes and managerial practices that may buffer the impact of large‐scale reforms on frontline nurses.

### 4.1 Limitations

However, several limitations should be acknowledged. Considering the operationalisation and feasibility of data collection, some potentially important variables, such as objective workload indicators (e.g., nurse‐to‐patient ratio and overtime), organisational and leadership characteristics and the frequency or intensity of organisational changes, were not included. Future studies could further examine these factors and explore their potential mediating mechanisms in the relationship between change fatigue and job crafting. Additionally, the cross‐sectional design limits causal interpretation of the observed associations. The generalisability of the findings may be limited, as the sample was drawn from hospitals in Hubei Province and primarily reflects the regional and organisational context of the local healthcare system. Despite these limitations, the large sample size provides useful insight into change fatigue and job crafting among Chinese nurses within a specific contextual setting.

## 5 Conclusion

Change fatigue and job crafting among nurses in Hubei Province, China, were found to be moderately high and negatively correlated. Additionally, factors such as age, type of hospital, professional title, income satisfaction, health condition and experience of changing employment were identified as predictors of change fatigue among nurses. In light of these findings, nursing managers should actively adopt individualised training, participatory leadership, sound communication and feedback mechanisms, and necessary resource support to actively prepare for organisational change, alleviate nurses' change pressure and fatigue, enhance nurses' subjective initiative and job crafting capabilities and foster an improved occupational environment conducive to both nurses' well‐being and the sustainable development of the organisation.

## 6 Implications for Practice

Clinical nurses have had to face and accept a variety of changes to adapt to the rapid changes in the healthcare field. While change improves the quality of nursing services, a series of negative impacts on nurses should not be underestimated. Therefore, based on this rapidly changing context, understanding nurses' change fatigue, job crafting and related factors is of great significance to nursing management. In this study, we investigated more than 20,000 nurses in Hubei Province, China, through a multicentre cross‐sectional study to clarify the level and correlation between change fatigue and job crafting of nurses and analyse the related factors of change fatigue, so as to provide targeted suggestions and strategies for improving nursing change fatigue. By understanding nurses' change fatigue and job crafting, it can help nursing managers to better plan and implement change management strategies, reduce the negative influence of change and improve the effectiveness and success rate of change management, as well as prevent and reduce nurses' work fatigue, improve the working environment, nursing quality and service level and realise the sustainable development of healthcare organisations.

## Author Contributions

**Xinyue Zhang:** conceptualisation; data acquisition; data analysis; data interpretation; writing – original draft preparation. **Ying Wang:** data interpretation; writing – review and editing; funding acquisition; supervision. **Yue Guo:** conceptualisation; investigation. **Shiyu Yin:** conceptualisation; investigation. **Zijing Huang:** writing – review and editing; visualisation. All authors have approved the final version for submission.

## Funding

This work was supported by Tongji Hospital (Grant 2022C08).

## Disclosure

*Statistics statement*: The authors affirm that the methods used in the data analyses are suitably applied to their data within their study design and context, and the statistical findings have been implemented and interpreted correctly.

## Ethics Statement

This study was approved by the Ethics Committee of Tongji Hospital, Tongji Medical College, Huazhong University of Science and Technology (No. TJ‐IRB202401089). The objectives and procedures were explained to the participants. They were assured that participation in the study was voluntary and anonymous and that refusing to participate would not have any impact on their work and life. All data collected is kept secure and confidential, accessible only to the research team.

## Conflicts of Interest

The authors declare no conflicts of interest.

## Data Availability

The data used in the study are available from the corresponding author upon reasonable request.
